# The role of adjuvant pharmacotherapy with liraglutide for patients with inadequate weight loss following bariatric surgery

**DOI:** 10.1007/s00423-023-02805-8

**Published:** 2023-03-03

**Authors:** James R. M. Colbourne, Oliver M. Fisher, Shirley Mo, Georgia S. Rigas, Michael L. Talbot

**Affiliations:** 1 Upper GI Surgery Unit, St George Hospital, Sydney, Australia; 2 Faculty of Medicine, University of Sydney, Sydney, Australia; 3 School of Surgery, University of Western Australia, Perth, Australia; 4 St George and Sutherland Clinical School - UNSW Medicine, University of New South Wales, Sydney, Australia

**Keywords:** Liraglutide, Bariatric surgery, Inadequate weight loss

## Abstract

**Purpose:**

Despite the benefits of bariatric surgery for many patients, there are a proportion of patients who do not achieve adequate weight loss. We evaluate the role of liraglutide as adjuvant pharmacotherapy in those who respond poorly to weight loss surgery.

**Materials and Methods:**

A non-controlled, prospective, open-label cohort study in which participants are prescribed liraglutide following inadequate response to weight loss surgery. The efficacy and tolerability of liraglutide was measured through measurement of BMI and monitoring of side effect profile.

**Results:**

A total of 68 partial responders to bariatric surgery were included in the study, 2 participants were lost to follow-up. Overall 89.7% lost weight on liraglutide, with 22.1% showing a good response (>10% total body weight loss). There were 41 patients who discontinued liraglutide mainly due to cost.

**Conclusion:**

Liraglutide is efficacious in achieving weight loss and reasonably well tolerated in patients who have inadequate weight loss post-bariatric surgery.

## Introduction

Obesity is a major worldwide problem, with an estimated 39% of adults considered overweight and a further 13% of adults who are obese. Obesity is a significant contributor to disease burden and is a major risk factor for many conditions, including type 2 diabetes, cardiovascular disease and certain malignancies. In OECD countries, healthcare and non-healthcare related costs are estimated to represent around 3.3% of total GDP [1]. Several medical and surgical options are available for weight loss. Of the surgical options, robust long-term data exist indicating sustained weight loss following the procedure, as well as a survival benefit, particularly among patients with diabetes [2, 3]. However, despite the benefits of weight loss surgery, a proportion of patients experience weight regain or inadequate weight loss after bariatric surgery [4]. Estimating the number of patients with inadequate weight loss following surgery is complicated by the heterogeneity of definitions in the literature and a paucity of high-quality data. Inadequate weight loss, however, remains a leading indication for revisional bariatric procedures and presents a unique challenge for both clinicians and patients [4, 5].

Several pharmacological solutions have been proposed for patients with inadequate weight loss or weight regain following bariatric surgery, including the glucagon-like-peptide-1 (GLP-1) receptor antagonist liraglutide. Liraglutide is generally safe and efficacious in the treatment of obesity, though patients who had undergone bariatric surgery were excluded from the original trials [6]. Emerging evidence suggests that liraglutide may be effective as a weight loss adjunct in patients who have had prior bariatric surgery; however, the effects of this treatment have not been studied specifically in patients with inadequate weight loss following bariatric surgery [7–9].

In this cohort study, we evaluated the efficacy and tolerability of liraglutide as an adjunct pharmacotherapy in patients with a partial response to bariatric surgery.

## Methods

### Study design, patients and treatment

A retrospective analysis was undertaken of a non-controlled patient cohort offered open-label liraglutide pharmacotherapy as an adjuvant in the event of inadequate weight loss (partial response) following bariatric surgery. In addition to weight loss, the tolerability and safety of liraglutide were evaluated in this population. Participants were aged 18–70 years and were recruited from patients who attended a routine post-bariatric surgery follow-up at a specialist clinic in Sydney, Australia. Ethics approval was granted by Ramsay Health Care NSW HREC (reference number: 2020-012).

Patients were offered at least 3 months of pharmacotherapy to achieve further weight loss. Included were patients at least one year post-laparoscopic sleeve gastrectomy (LSG) or Roux-en-Y gastric bypass (RYG) or at least 2 years post-laparoscopic gastric band (LGB) and had inadequate weight loss defined as BMI >35 (all patients), > 25% TWL (LSG, RYGBP), >20% TWL (LAGB), and in patients who self-identified as weight stable after surgery without meeting health or weight goals. As patients were required to self-fund therapy, they were a group who were motivated to lose more weight but had not achieved it with their chosen therapy. Patients with a surgical reason for weight regain, reported pregnancy, ischaemic heart disease, cardiac conduction disorders or contraindication to liraglutide were excluded.

Eligible patients were prescribed liraglutide using the dose-escalation protocol (1.8–3.0 mg subcut daily) outlined by the manufacturer [10]. Patients who did not lose at least 5% of their starting weight after 3 months of treatment were deemed non-responders to pharmacotherapy, and therapy was ceased.

### Assessment and statistical analysis

Data were collected from patient electronic medical records, and descriptive statistics were reported, including averages as median with interquartile range or mean, where appropriate. Data were compared using Fisher’s exact test for categorical or a two-sample *t*-test for parametric data or the Wilcoxon signed-rank test/Mann–Whitney *U* test for non-parametric data. All pre versus post treatment analysis were performed as paired calculations. A univariable logistic regression was conducted to identify the factors that may have contributed to a good response to liraglutide. Significant factors identified following univariable analysis were subsequently used in the multivariable logistic regression modelling. The results were considered statistically significant if *p* < 0.05. All statistical analyses were conducted using R [11].

## Results

Out of a larger, 291-participant study evaluating the outcomes of adjuvant pharmacotherapy with liraglutide, a total of 68 participants were identified as partial responders to bariatric surgery and included in the final study cohort. Demographic data, participants’ medical backgrounds and safety and tolerability data were collected and available for all participants. The median age of participants was 40.5 years (34.3–47.8 years), and 57 participants (83.8%) were female. At the time of commencement of liraglutide, four (5.9%) patients had diabetes, 35 (51.5%) had been diagnosed with insulin resistance, 21 had hypertension (30.9%), 24 (35.3%) had hyperlipidaemia, 15 (22.1%) self-reported having a mental illness and 12 (17.6%) had thyroid disease (Table 1).

**Table 1:** Baseline patient demographics prior to liraglutide treatment

Characteristic	Patients (*n*=68)
Sex, *n* (%)	
Female	57 (83.8%)
Male	11 (16.2%)
Age at surgery, years	
Median	40.5
Range	34.3–47.8
Age at weight nadir, years	
Median	45.0
Range	39.0–51.0
Time to weight nadir, months	
Median	15.5
Range	0–88.3
Comorbidities	
Diabetes	4 (5.9%)
Insulin resistance	35 (51.5%)
Hypertension	21 (30.9%)
Obstructive sleep apnoea	12 (17.6%)
Hyperlipidaemia	24 (35.3%)
Mental illness	15 (22.1%)
Thyroid disease	12 (17.6%)
Types of bariatric surgery	
Laparoscopic sleeve gastrectomy	30 (44.1%)
Laparoscopic gastric band	18 (26.5%)
Roux-en-Y gastric bypass	15 (22.1%)
Other	5 (7.4%)
Revisional bariatric surgery patients	20 (29.4%)

Most patients (30; 44.1%) had undergone a previous laparoscopic sleeve gastrectomy, 18 (26.5%) had a gastric band, 15 (22.1%) had undergone RYG bypass and five (7.4%) underwent other types of bariatric surgeries. A total of 20 (29.4%) patients had revisional bariatric surgery procedures prior to commencing liraglutide (Table 1).

Forty-one (60.2%) patients discontinued liraglutide, with 38 of those doing so in the first 6 months. The main reason for discontinuing the therapy cited by patients was cost (52.5%), followed by side effects (19.5%) and being content with their weight (17.1%). Eight (11.8%) patients reported side effects but no serious adverse events, with abdominal pain and nausea being the most frequently reported effects (Table 2).

**Table 2:** Side effect profile and tolerability of liraglutide treatment

Characteristic	Patients (*n*=68)
Patients who discontinued liraglutide	41 (60.2)
Time to discontinuation	
4 months	16
6 months	22
9 months	2
12 months	1
Reasons for discontinuation	
Cost	20 (52.5%)
Side effects	8 (19.5%)
Content with weight	7 (17.1%)
Lost to follow-up	2 (4.9%)
Not specified	4 (9.8%)
Side effects liraglutide	
Reported side effects of unspecified nature	8 (11.8%)
Abdominal pain	4 (5.9%)
Constipation	1 (1.5%)
Nausea	3 (4.4%)
Palpitations	1 (1.5%)
Lethargy	1 (1.5%)
Rash	1 (1.5%)

**Fig. 1:**
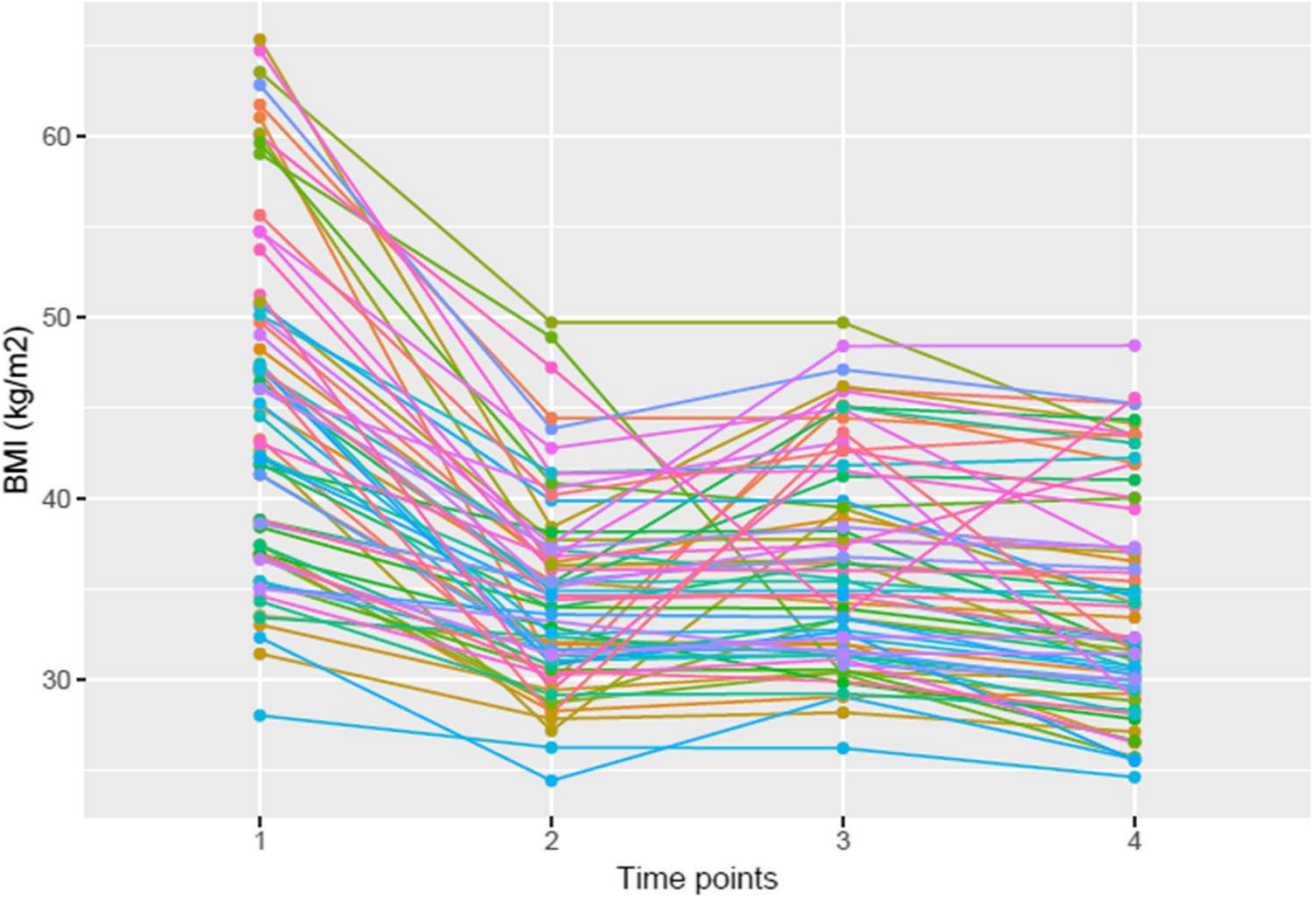
Plot of change in body mass index (BMI) at intervals during trial. (1) Prior to bariatric surgery (68/68 patients); (2) post-surgery nadir (68/68 patients); (3) prior to commencement of liraglutide (68/68 patients); (4) last documented BMI in study (66–68 patients)

**Fig. 2:**
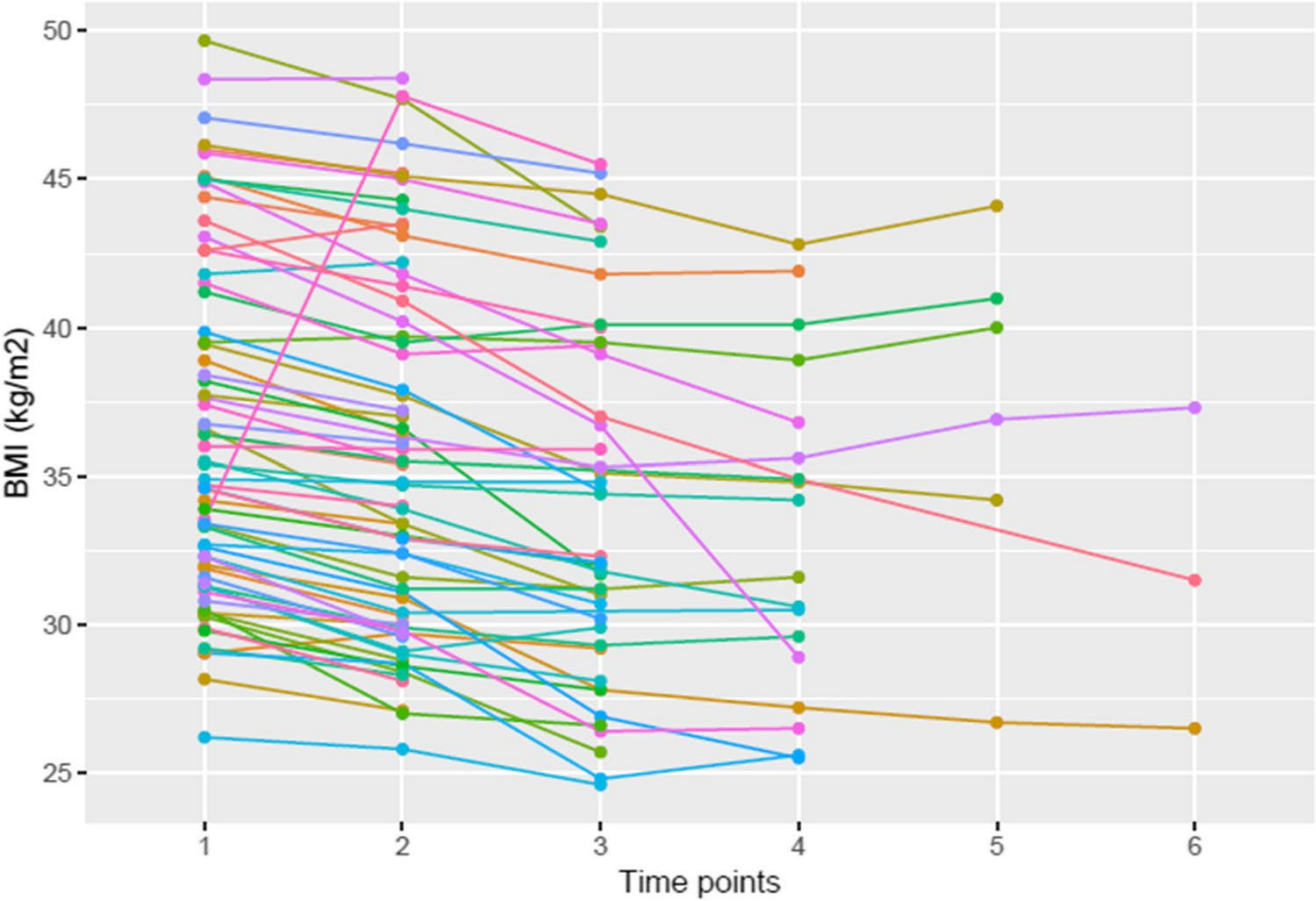
Plot of change in body mass index (BMI) at various follow-up intervals during liraglutide treatment. (1) pre-treatment BMI (68/68 patients); (2) BMI at 1 month (68/68 patients); (3) BMI at 4 months (68/68 patients); (4) BMI at 7 months (66/68 patients); (5) BMI at 10 months (66/68 patients); (6) BMI at 13 months (66/68 patients)

The median BMI prior to surgery was 44.8 (37.1–50.5) and median BMI nadir was 34.6 (30.8–37.3) corresponding to a median weight prior to surgery of 117.4 (100.6–142.6) and weight nadir of 89.1 (82.5–105.7). The median time to weight/BMI nadir was 15.5 months (0–88.25). Prior to commencing treatment with liraglutide, the median BMI was 35.2 (31.6–41.3), and median weight was 95.8 (93.4–117.6). At follow-up, the BMI and weight reduced to 32.3 (29.8–39.7) and 90.3 kg (78.45–113.1), respectively (Figs. 1–2, *p*<0.001). This corresponded to a median total body weight loss (TBWL) of 5.30% (2.23% to 8.35%, Table 3).

**Table 3:** Weight and body mass index (BMI) change in response to liraglutide treatment

Characteristic, average	Patients (*n*=68)	*p*-value
Average BMI (kg/m^2^)		
Prior to bariatric surgery	44.8 (37.1–50.5)	*p* < 0.001
At weight nadir	34.6 (30.8–37.3)	
Prior to liraglutide start	35.2 (31.6–41.3)	*p* < 0.001
After liraglutide	32.3 (29.8–39.6)	
Change	1.85 (0.85–3.25)	*p* < 0.001
Average weight (kg)		
Prior to bariatric surgery	117.4 (100.6–142.6)	*p* < 0.001
At weight nadir	89.1 (82.5–105.7)	
Prior to liraglutide start	95.8 (93.4–117.6)	*p* < 0.001
After liraglutide	90.3 (78.5–113.1)	
Change	5.2 ( 2.2–6.6)	*p* < 0.001
Patients who lost weight on liraglutide	61 (89.7%)	
Patients who responded well to liraglutide (>10% EBWL)	15 (22.1%)	
% total body weight loss on liraglutide	5.30% (2.23–8.35%)	
% total body weight loss by bariatric surgery		*p* < 0.031
Laparoscopic sleeve gastrectomy	3.63% (2.02*–*6.11)	
Laparoscopic gastric band	4.76% (0.84–9.22)	
Roux-en-Y gastric bypass	6.39% (5.30–13.0)	
Other	5.76% (1.91–5.89)	

Of the study participants, 61 (89.7%) lost weight on liraglutide and 15 (22.1%) showed good response (≥10% TBWL) to liraglutide. Revisional bariatric surgery was the only factor found to contribute to weight loss following liraglutide: patients who had undergone revision bariatric surgery had higher TBWL on average (median 6.50%) than those who had not undergone a revision procedure (median 4.10%, *p*=0.021). No other demographic factors or comorbidities were identified as being associated with weight loss following use of liraglutide upon uni- and multivariable regression analysis.

## Discussion

This open-label cohort study was conducted to examine the effect and tolerability of liraglutide in patients who exhibit a partial response to bariatric surgery. The main finding was that partial responders to bariatric surgery who were prescribed liraglutide 3.0mg subcut daily lost on average 5.30% (2.23% to 8.35%) of their total body weight loss, corresponding to a decrease in BMI from 35.2 (31.6–41.3) to 32.3 (29.8–39.6). This positive finding was observed despite 60.2% of patients prematurely discontinuing treatment and 11.8% of patients overall experiencing one or more side effects.

To our knowledge, our study is the first to specifically consider the role of adjuvant pharmacotherapy liraglutide in patients who have a partial response to bariatric surgery and thus may be deemed to have inadequate weight loss. In this group of patients, the modest but clinically significant weight loss observed is indicative of a promising adjunctive pharmacotherapy option in what can sometimes be very challenging surgical and clinical circumstances.

Liraglutide functions as a glucagon-like-peptide (GLP 1) agonist, and several randomised controlled trials support its role as a weight loss agent [6, 12]. In these studies, the average weight change ranged from 7.2–8.4 kg in patients who had not previously had bariatric surgery, which is greater than the 5.2 kg weight change that we observed in our study. However, there were lower rates of discontinuation in these studies, which could be partly due to the drug being provided free of charge. Several trials have specifically considered liraglutide following bariatric surgery, research by including Wharton et al. (2019), which examined 117 patients prescribed 3.0 mg liraglutide and noted a 5.5% ± 6.2% TBWL, which corresponds well with our findings. However, the study included patients who had insufficient weight loss or excessive weight regain. In addition, the authors did not observe that the type of bariatric surgery made a difference in weight loss. Although we did note a significant difference between weight loss and the type of bariatric surgery, we only found the difference between gastric bypass procedures and adjustable gastric bands to be significant. However, the type of bariatric surgery was not predictive of a good response to liraglutide in our multivariable analysis. Other studies have demonstrated similar weight loss, although prior research has not specifically studied patients who show insufficient weight loss following bariatric surgery. In Rye et al.’s (2018) study on 20 patients, seven presented with inadequate weight loss, and the total cohort experienced 8.4% TBWL after 28 weeks (though patients who discontinued early were not included in the final analysis).

An important issue identified in this study was that of discontinuation of therapy. Forty-one (60.2%) patients discontinued therapy; of these patients, 32.3% discontinued the therapy after 6 months. The primary reasons given for this were cost, side effects and contentment with weight loss. In Australia, treatment costs around 390 AUD per month, and the therapy is not subsidised by the government’s Pharmaceutical Benefits Scheme. As such, patients are required to carry the full cost of treatment themselves and thus the discontinuation of treatment is not entirely surprising. Other studies that have provided the drug to patients have generally found a rate of premature discontinuation of less than 10%, indicating that cost is a significant barrier to be overcome [6, 12, 13].

Another common cause of discontinuation in the present study was the side effect profile of the drug. Overall, eight patients (11.8%) reported side effects, the most common of which were gastrointestinal symptoms, including abdominal pain and nausea. These side effects are widely reported, including in the post-bariatric surgery population [8, 13, 14]. It is reassuring that while participants reporting side effects also cited this as a reason for discontinuation, the absolute number of patients who experienced side effects was low, indicating reasonable tolerability.

Given the tolerability of liraglutide and its efficacy in inducing weight loss, this therapy could represent an alternative to revision bariatric surgery in patients with inadequate weight loss following primary surgery. Revisional bariatric surgery is associated with a significant risk of morbidity and mortality and, similar to pharmacotherapy, is not always successful [15–18]. Horber et al. (2021) studied the efficacy of liraglutide as an alternative to revisional surgery. The authors compared 95 patients with weight regain at least 6 years post-RYG and who had either received liraglutide, surgical revision or lifestyle modification advice. Here, the authors found that liraglutide was non-inferior to revision and was associated with a change in BMI of 4.8 ± 2.9 kg/m^2^. Although the population studied differs from our study population in that the participants were weight regainers rather than patients with inadequate weight loss, overall, the findings support the use of liraglutide.

The present study had several limitations including the lack of a control group, non-blinding and potential selection bias due to the open-label nature of the study. The non-randomized prospective trial design is limited in the ability to precisely calculate effect from intervention. Despite this, we believe the trial design does reflect what is seen in clinical practice. In addition, the relatively high rate of discontinuation of therapy may have influenced the overall assessment of weight change. As a high proportion of patients who discontinued therapy did so because of cost, future studies may wish to consider funding drug access to be able to better evaluate the true efficacy of the drug. However, cost can represent a significant barrier to patient medication compliance; therefore, this reason for discontinuation likely reflects a real-world scenario.

## Conclusions

Our study supports the use of liraglutide 3.0mg subcut daily as an adjunct to weight loss in patients with inadequate weight loss following bariatric surgery. This study builds on research demonstrating the efficacy of liraglutide in other post-bariatric surgery groups, including patients who have regained weight. We found that the treatment was reasonably well tolerated and efficacious, although we did not identify which patient factors illicit a good response to therapy. Further studies that include a control group could provide additional insight into the utility of liraglutide in this clinically challenging patient population.
